# Screening of 50 Cypriot Patients with Autism Spectrum Disorders or Autistic Features Using 400K Custom Array-CGH

**DOI:** 10.1155/2013/843027

**Published:** 2013-10-24

**Authors:** Ludmila Kousoulidou, Maria Moutafi, Paola Nicolaides, Stavros Hadjiloizou, Christos Christofi, Anna Paradesiotou, Violetta Anastasiadou, Carolina Sismani, Philippos C. Patsalis

**Affiliations:** ^1^The Cyprus Institute of Neurology and Genetics, P.O. Box 23462, 1683 Nicosia, Cyprus; ^2^The Cyprus Paediatric Neurology Institute, 2047 Nicosia, Cyprus; ^3^Archbishop Makareios III Hospital, 2012 Nicosia, Cyprus

## Abstract

Autism spectrum disorders (ASDs) comprise a distinct entity of neurodevelopmental disorders with a strong genetic component. Despite the identification of several candidate genes and causative genomic copy number variations (CNVs), the majority of ASD cases still remain unresolved. We have applied microarray-based comparative genomic hybridization (array-CGH) using Agilent 400K custom array in the first Cyprus population screening for identification of ASD-associated CNVs. A cohort of 50 ASD patients (G1), their parents (G2), 50 ethnically matched normal controls (G3), and 80 normal individuals having children with various developmental and neurological conditions (G4) were tested. As a result, 14 patients were found to carry 20 potentially causative aberrations, two of which were *de novo*. Comparison of the four population groups revealed an increased rate of rare disease-associated variants in normal parents of children with autism. The above data provided additional evidence, supporting the complexity of ASD aetiology in comparison to other developmental disorders involving cognitive impairment. Furthermore, we have demonstrated the rationale of a more targeted approach combining accurate clinical description with high-resolution population-oriented genomic screening for defining the role of CNVs in autism and identifying meaningful associations on the molecular level.

## 1. Introduction

Autism spectrum disorders (ASD) (OMIM 209850) comprise a group of neuropsychiatric developmental disorders, affecting approximately 1% of the general population [1]. The strong genetic component of ASD has been evident since early twin and family studies [2], while it is currently accepted that autism most likely results from a combination of genetic, epigenetic, and environmental factors [3].

Genome-wide association studies have linked multiple regions to autism, and the role of genetic alterations within several genes for example, *SHANK3, NRXN1, NLGN3, NLGN4X* and* CNTNAP2* [4, 5] is recognized as pathogenic. The contribution of genomic copy number variations (CNVs) has been extensively explored by screening patients using array-based comparative genomic hybridization (array-CGH), demonstrating the clinical relevance of *de novo* CNVs in syndromic and nonsyndromic autism [6, 7]. Despite the progress in the identification of new potentially causative CNVs, the vast majority of autism cases still remains unexplained. An obstacle for autism genotype-phenotype correlations has been clinical and genetic heterogeneity of patient cohorts in combination with an apparently multi-genic determinant of the disease, making it difficult to connect a single gene with a distinct clinical feature. 

We have limited our sample heterogeneity by focusing on the small and genetically homogenous population of Cyprus and applied a robust CNV detection technique, namely Agilent 400K custom array-CGH. This platform combines high resolution with relatively low complexity of analysis and can reliably identify deletions and duplications as small as 13 kb. Genetic screening was directed towards four different groups of population: 50 selected patients with ASD or autistic features, their parents, 50 ethnically matched normal controls, and 80 normal individuals having children with syndromic or nonsyndromic mental retardation, developmental delay, or rare neurological syndromes. With the assumption that ASDs are underdiagnosed or misdiagnosed in the Cypriot population, we have also performed a clinical reevaluation of a group of patients who were given a preliminary diagnosis of “ASD”. 

## 2. Materials and Methods

We studied four different groups from the population of Cyprus. Group 1 (G1) includes 50 children (45 boys and 5 girls) 3–18 years of age at the time of recruitment, with a preliminary diagnosis of autism spectrum disorders (ASD). There was no gender-based selection of the participants and the prevalence of males in G1 reflects the general male to female ratio among individuals with ASD; group 2 (G2) includes the nonaffected biological parents of the G1 children (with the exception of the father of patient 11, who has mild mental retardation and autistic features); group 3 (G3) includes a control cohort of 50 normal participants (18 males and 32 females), selected to be older than 30 years of age and have at least two biological children with no mental, neurological, or developmental dysfunction; group 4 (G4) includes 80 normal individuals having children with syndromic or nonsyndromic mental retardation, developmental delay, or rare neurological syndromes. The normal individuals of G4 had participated in previous screening studies, focused on array-CGH testing of their affected children (unpublished data). All patients were specifically selected to have normal karyotype and be negative for fragile-X syndrome. 

All patients from G1 were reevaluated by a clinical geneticist to rule-out autistic-like syndromes and retested for ASD based on the Diagnostic and Statistical Manual of Mental Disorders for Physician, Text Revision (DSM-IV-TR) and International Classification of Diseases, Tenth Revision (ICD-10), using Gilliam Autism Rating Scale-2 (GARS-2). 

DNA was extracted with Qiagen DNA extraction kit (Qiagen Co) according to the manufacturer's recommendations. Array-based comparative genomic hybridization (array-CGH) was performed using standard protocols with 400K oligonucleotide custom array platform (Agilent Santa Clara, CA). This array includes the entire 4 × 180 K ISCA (International Standard Cytogenomic Array) design which has a strong clinical emphasis and covers 9269 polymorphic regions derived from the Wellcome Trust case control consortium CNV genotyping array, mainly based on the 42 M array data. In addition, the array includes another 209214 probes providing a backbone coverage of 13 kb.

Image analysis, normalization, and annotation were based on Agilent Feature Extraction 9.1, while Nexus Copy Number 5.1 software (BioDiscovery Inc.) was applied for visualization of data, data analysis, and filtering. The main criteria applied to assess the potential causality of detected CNVs were (i) localization within a gene or gene-rich region, (ii) less than 80% overlap with known benign CNVs and with CNVs present in G3, (iii) overlap with syndrome regions described in Decipher database (http://decipher.sanger.ac.uk/), (iv) assessment of overlapping gene function with special attention to genes, known for their role in ASD [5], and (v) *de novo* occurrence was taken into account; however, inherited aberrations that fit criteria (i)–(iv) were not excluded. 

All array-CGH findings presented in Table 1 were confirmed with Quantitative Real-Time PCR (QRT-PCR) using two sets of primers for each region. Primers were designed with primer 3 software and ran through an *in silico* PCR to check for secondary amplification sites. QRT-PCR was carried out with 10 ng of genomic DNA in 10 *μ*L reaction including 5 *μ*L of SsoFast EvaGreen supermix (Bio-Rad), and 3 *μ*L of 0.5 *μ*M primer (Metabion). All reactions were performed in triplicate in 96-well plates (Bio-Rad) and ran on CFX96 Real-Time System C1000 Thermal Cycler (Bio-Rad). 

## 3. Results

Clinical reevaluation of the patients has shown that only 23 out of the 50 participants can be accurately characterized as having nonsyndromic ASD, while the remaining 27 patients have autistic features accompanying mental retardation, developmental or psychomotor delay, epilepsy, and dysmorphism. 

**Figure 1:**
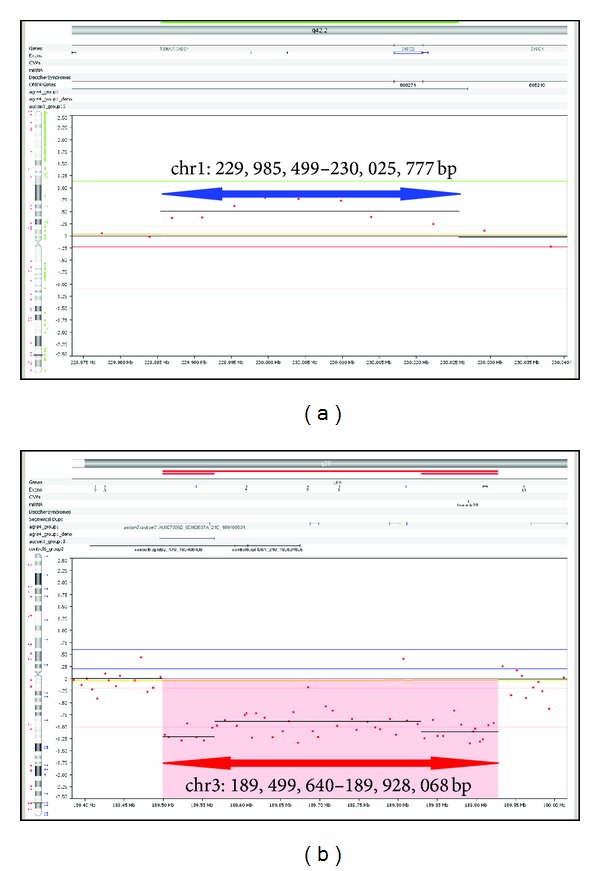
Array-CGH profiles, highlighting the two *de novo* aberrations: (a) 40.27 kb duplication on 1q42.1 and (b) 428.4 kb deletion on 3q28.

400K array-CGH resulted in the detection of twenty potentially causative aberrations, in fourteen patients (Table 1). The majority of these aberrations was also present in the parents (G2), with only two *de novo* events in two different patients including a 40.27 kb duplication on 1q42.1 (patient 2, Figure 1(a)) and a 428.4 kb deletion on 3q28 (patient 12, Figure 1(b)). Duplications overlapping with the 1q42.1 CNV were found in eight normal individuals of G4 and two normal individuals from G3. A 3q29 deletion in patient 11 was inherited from an affected father and is associated with autistic features. Two of the twenty aberrations (patients 1 and 5) are found in copy number variation databases; one aberration was found in both G3 and G4 (patient 2), and one was found in G3 (patient 13). Eighteen aberrations reside within genes, implicated in autism susceptibility, and two are associated with mental retardation and developmental delay (Table 1). 

## 4. Discussion

We present the first investigation of the genetic basis of ASD, carried out for the population of Cyprus. Unlike widely performed large-scale screening studies, we opted to focus on this relatively small and genetically homogenous population in order to maximize the probability of association given a smaller cohort size. Moreover, we included three different ethically matched control groups with the aim to gain more insight into variable expressivity and comorbidity aspects, both of which play an important role in ASD. Thus, even though the number of studied individuals is not large enough to draw statistically significant conclusions, the genetic homogeneity of the participants allowed for some cautious assumptions based on general observations.

One of the challenges encountered in this study was the accuracy of ASDs diagnosis, clearly differentiating them from syndromic neurological conditions, developmental delay, or mental retardation with autistic features. The reevaluation of our patients (G1) has demonstrated that clinical genetic assessment is a vital addition to neurological, psychiatric, or psychological evaluation as a tool for ruling-out genetic syndromes that may affect development and mimic the clinical manifestation of ASD. 

In Table 1, we have used the publication by Pinto et al. [5] as reference for autism-associated variants. Such variants were detected in patients with syndromic conditions that included autistic features, for example, patients 4 and 10. Patient 10, who has inherited a deletion within *CNTP2* gene from his unaffected mother, received a preliminary diagnosis of ASD at the age of two years. Screening for metabolic disorders and MRI investigation were negative and no family history has been reported. He suffers from social communication disorder, speech impairment, learning difficulties, global developmental and psychomotor delay, stereotyped behavior, and hyperactivity. In line with the widely observed comorbidity of ASD, the presence of autistic features in the clinical picture even as part of a syndrome, may point to an autism-associated genetic cause. Likewise, the detection of CNVs linked to mental retardation and/or developmental delay is common in patients with nonsyndromic ASD and is demonstrated in our study (e.g., patients 2 and 9). This phenomenon can be explained by shared pathways related to key brain functions; however, the exact cause and timing of differentiation between the different phenotypic outcomes is currently unknown. 

The *de novo* deletion in patient 12 and the inherited deletion in patient 13 (also found in G3) both include miRNA coding genes, so their effects may be mediated by the transcriptional silencing of these miRNAs towards their targets. Identification of the miRNA targets would help understand the molecular mechanism that lead to the manifestation of autistic features in these patients. 

The *de novo* 40.27 kb duplication detected in patient 2 encompassing *DISC1* and *DISC2* genes, known for their role in ASD [8], is present in G3 and G4 with two and eight normal carriers, respectively. Patient 2 has a typical ASD phenotype with speech impairment, communication difficulties, poor eye contact, behavioral problems, and hyperactivity. Magnetic resonance imaging (MRI) and electroencephalography (EEG) revealed no abnormalities, and his family history is unremarkable. We assume that this duplication does not have the strong pathogenic effect of the previously reported deletions within this region and may be a population-specific variant, possibly predisposing to ASD under certain conditions. 

The above data support the multifactorial model of autism, where potentially causative aberrations exhibit variable penetrance depending on the genetic background, often defined as the synergy of other coexisting aberrations [9]. In our study, some patients (patients 5, 7, 8, 10, 11, and 14) were found to carry more than one potentially causative CNV. In the case of patient 11, the deletion within the known 3q29 microdeletion syndrome region inherited form an affected father is most probably the primary cause of the child's phenotype, which includes learning difficulties, poor concentration (without hyperactivity), and mild intellectual disability with normal EEG. Patient 8 inherited two aberrations from his mother, one of which affects the known 1q21.1 microdeletion/duplication syndrome region. He has a relatively mild ASD phenotype and attends a private secondary school with some educational support. Despite his normal speech, he is socially isolated form his peers, exhibits stereotyped hand movements, reduced facial expressivity, and emotional withdrawal, echolalia, psychomotor hyperactivity, and narrow interests. His mother suffers from depression after being disabled due to an accident. The hypothesis of a combined causative effect is more applicable to the cases where the CNVs are inherited from different parents (as in patients 10, 11, and 14) rather than from a single healthy carrier (patients 5, 7, and 8). At the same time, it should be noted that the resolution of 400K array-CGH cannot rule-out smaller contributing aberrations, and the data interpretation cannot exclude the possibility of potentially significant findings being filtered out as benign. Moreover, one cannot ignore the effect of possible epigenetic and environmental modifications, adding more complexity to the disease aetiology. 

Finally, we have observed significant differentiation of G2 (parents of ASD patients) from other nonaffected parent groups in terms of the number of CNVs within genes that are associated with ASD. Six out of fifty mothers and 8/50 fathers from a total of 100 parents (14%) appear to carry 16 different rare variants associated with ASD or MD/DD, not found in international CNV databases nor in any of the studied Cypriot groups. In contrast, the difference between G3 and G4 cannot be considered significant as it concerns only one aberration, which is likely to be a population-specific variant. The presence of such “genetic load” within G2 is an addition to the mounting evidence concerning the role of multiple (genetic and nongenetic) factors in the occurrence of autism or autistic features. 

## 5. Conclusions

In this study, we addressed some important issues relevant to the genetic characterization of ASDs, such as differential diagnosis, cohort homogeneity, variable expressivity, and comorbidity. 

400K array-CGH analysis of our patient cohort along with three ethnically matched control groups has provided supporting evidence about the complexity of ASD aetiology in comparison to other developmental disorders involving cognitive impairment. 

Our data have demonstrated that a more targeted approach combining accurate clinical description with high-resolution population-oriented genomic screening is a promising strategy for defining the role of CNVs in autism and identifying meaningful associations on the molecular level. Further studies are required in order to reveal the total of genetic and environmental factors that lead to the disease. 

## Figures and Tables

**Table 1:** List of the detected candidate autism susceptibility aberrations.

Patient	Phenotype	Gender	Event	Size	Chr. Position hg18	Inheritance	Genes	CNVs	Decipher	ASD regions [5]	In G2 %	In G3 %	In G4 %
1	Autism	F	Del	27.16 kb	chr19:59,840,527-59,868,239	Father	*LILRB1, LILRB4 *	YES	AUTISM	YES	1	0	0

2	ASD	M	Dup	40.27 kb	chr1:229,985,499-230,025,777	**de novo**	*TSNAX1- DISC1, DISC1, DISC2 *	NO	MR/DD	YES	0	4	10

3	DD, DF, AF	M	Dup	304.87 kb	chr5:61,885,191-62,190,063	Father	*IPO11, LRRC70 *	NO	MR/DD	YES	1	0	0

4	MR, epilepsy, AF	M	Dup	829 kb	chr11:132,447,787-133,276,750	Father	*OPCML *	NO	AUTISM/SPEECH DELAY	YES	1	0	0

5	Autism	M	Del	167.4 kb	chr2:212,521,344-212,688,773	Mother	*ERBB4 *	NO	SPEECH DEFECTS	YES	1	0	0
			Del	191.3 kb	chr3:60,047,028-60,238,353	Mother	*FHIT *	YES	SPEECH DELAY/MR/DD	YES	1	0	0

6	ASD, MR	M	Dup	101.78 kb	chr17:9,906,791-10,008,572	Father	*GAS7 *	NO	NO	YES	1	0	0

7	ASD high functioning	M	Dup	923.6 kb	chr22:47,373,766-48,297,411	Mother	*FAM19A5 (TAFA5) *	NO	AUTISM	YES	1	0	0
			Del	41.87 kb	chr2:178,247,791-178,289,664	Mother	*PDE11A *	NO	SPEECH DELAY	YES	1	0	0

8	ASD high functioning	M	Del	101.4 kb	chr3:6,981,830-7,103,523	Mother	*GRM7 *	NO	SPEECH DELAY	YES	1	0	0
			Dup	2.24 Mb	chr1:144,513,497-146,753,802	Mother	26 GENES	NO	MICRODEL/DUP SYND	YES	1	0	0

9	ASD	M	Del	77.79 kb	chr9:9,598,565-9,691,922	Father	*PTPRD *	NO	MR/DD, ADHD	YES	1	0	0

10	Psychomotor delay, AF	M	Del	25.45 kb	chr7:146,067,147-146,092,598	Mother	*CNTNAP2 *	NO	AUTISM	YES	1	0	0
			Dup	41.69 kb	chr16:8,715,900-8,757,596	Father	*ABAT* (GABA METABOLISM)	NO	MR/DD	NO	1	0	0

11	MR, AF	M	Del	1.07 Mb	chr3:197216353-198287118	Affected father	numerous	NO	3q29 microdeletion syndrome	YES	1	0	0
			Del	132 kb	chr4:124119791-124251951	Mother	*SPATA5 *	NO	WITH MR	YES	1	0	0

12	MR, AF	F	Del	428.4 kb	chr3:189499640-189928068	**de novo**	*LPP, hsa-mir-28 *	NO	MR/DD	YES	0	0	0

13	ASD	M	Del	142 kb	chr9:28719400-28861226	Father	*has-mir-876 *	NO	AUTISM/MR/DD	YES	1	0.5	0

14	MR, ASD	M	Dup	242 kb	chr4:74032829-74275631	Mother	*ANKRD17, OX18, COX18HS *	NO	SPEECH DELAY	YES	1	0	0
			Del	96.5 kb	chr1:239,357,689-239,454,247	Father	*MIR3123, RGS7* (neuronal exitability)	NO	MR/DD	NO	1	0	0

ASD: autism spectrum disorder; MR: mental retardation; DD: developmental delay; AF: autistic features; DF: dysmorphic features; CNV: copy number variation.
